# Long-term maintenance of a *Deltacoronavirus* infecting multiple bird species in Antarctica

**DOI:** 10.1128/spectrum.02688-24

**Published:** 2025-06-16

**Authors:** Fernanda Gomes, Alexandre Freitas da Silva, Tatiana Prado, Paola Cristina Resende, Leonardo Corrêa Da Silva Junior, Wim Degrave, Maithê Magalhães, Adriana Vivioni, Yago José Mariz Dias, Marilda Siqueira, Martha Brandão, Luciana R. Appolinario, Gabriel da Luz Wallau, Maria Ogrzewalska

**Affiliations:** 1 Laboratory of Respiratory, Exanthematic, Enteric Viruses and Viral Emergencies, Oswaldo Cruz Institute, Oswaldo Cruz Foundation 37903, Rio de Janeiro, Brazil; 2 Departamento de Entomologia, Instituto Aggeu Magalhães (IAM)—Fundação Oswaldo Cruz (Fiocruz) 92923 , Recife, Brazil; 3 Núcleo de Bioinformática, Instituto Aggeu Magalhães (IAM)—Fundação Oswaldo Cruz (Fiocruz) 92923 , Recife, Brazil; 4 Laboratory of Applied Genomics and Bioinnovation, Oswaldo Cruz Institute, Oswaldo Cruz Foundation 37903, Rio de Janeiro, Brazil; 5 Laboratory of Bacteriology applied to One Health and Antimicrobial Resistance, Oswaldo Cruz Institute 196605 , Rio de Janeiro, Brazil; 6 Vice-Presidency of Production and Innovation in Health, Oswaldo Cruz Foundation 37903, Rio de Janeiro, Brazil; 7 Department of Arbovirology and Entomology, WHO Collaborating Center for Arbovirus and Hemorrhagic Fever Reference and Research, National Reference Center for Tropical Infectious Diseases, Bernhard Nocht Institute for Tropical Medicine 14888, Hamburg, Germany; 8 Programa de Pós-graduação em Biodiversidade Animal and Programa de Pós-graduação em Bioquímica Toxicológica, Universidade Federal Santa Maria (UFSM) 28118, Santa Maria, Brazil; Erasmus MC, Rotterdam, the Netherlands

**Keywords:** active surveillance, Antarctic wildlife, viral infections, zoonotic risks, environmental fecal samples

## Abstract

Antarctica’s pristine environment and wildlife are of immense biological value, but the spread of viruses poses a substantial threat to the region’s fragile ecosystems. To investigate the presence of viruses in the region, we sampled the feces of different wild migratory birds at various localities in the South Shetland Islands and screened them for coronaviruses (CoVs) and avian influenza viruses (AIVs). Viral screening was performed by the conventional pancoronavirus RT-PCR protocol (CoVs), by one-step real-time RT-PCR (AIVs) followed by metatranscriptomic sequencing of positive samples. During January and February of 2023, we collected and examined 243 fecal samples representing skuas (*Stercorarius* spp.), kelp gulls (*Larus dominicanus*), Antarctic shags (*Phalacrocorax bransfieldensis*), Adélie penguins (*Pygoscelis adeliae*), chinstrap penguins (*Pygoscelis antarcticus*), gentoo penguins (*Pygoscelis papua*), and penguins of the *Pygoscelis* genus. All tested samples were negative for AIVs, and one sample from the colony of *L. dominicanus* tested positive for CoVs. Metatranscriptomic sequencing recovered a full *Deltacoronavirus* genome. Nucleotide and amino acid distance analysis revealed an unratified species of the genus *Deltacoronavirus* belonging to the subgenus *Buldecovirus* and to a previously proposed novel wild bird *Deltacoronavirus* clade previously identified in 2014 in *P. papua* penguin. The detection of the same virus infecting multiple bird species in Antarctica suggests a potential cross-species transmission or that this virus has a broad host range naturally infecting multiple bird species. The presence of CoVs in Antarctic migratory seabirds does not necessarily indicate a concern, as many of these viruses may have co-evolved with their hosts and circulate without causing clinical disease. However, continued monitoring of the Antarctic bird fauna, particularly focused on diseased animals and their association with this virus, warrants further investigations. This new finding brings valuable insights into virus dynamics among seabird populations, laying the groundwork for future investigations in this field and warning for the importance of viral surveillance on the Antarctic fauna.

**IMPORTANCE:**

Antarctica’s ecosystems are highly vulnerable to external threats, and the spread of viruses could significantly impact the region’s wildlife. This study emphasizes the importance of monitoring migratory seabirds, as they can serve as vectors for viral transmission and eventually disease, including coronaviruses and avian influenza viruses. By detecting *Deltacoronavirus* in kelp gull feces, which was previously detected in a penguin, this research highlights the potential for cross-species transmission or multi-host infection capacity for this virus. These findings stress the need for increased viral surveillance in the Antarctic region, which could help protect local wildlife and provide essential data for understanding viral dynamics and infection in natural settings. Proactive monitoring will be crucial for mitigating the risk of zoonotic outbreaks and maintaining the integrity of Antarctica’s fragile ecosystems.

## INTRODUCTION

Antarctica harbors a rich and unique endemic fauna, including several bird species (penguins, petrels, cormorants, gulls, skuas, terns), along with its associated microbiome (1). The geographic isolation of this continent, coupled with the extreme climate and unique ecological interactions, has historically been assumed to protect the indigenous Antarctic wildlife from exposure to infectious agents (2). However, in recent decades, this pristine environment has been increasingly threatened by human activities, such as tourism, marine exploitation, and waste disposal, which compromise ecosystem health (3–5). The introduction of exotic or non-native species, including the introduction of new pathogens, alongside climate change, is an additional factor contributing to ecological changes that have the potential for population reductions in endangered species due to mortality (5–7). While surveillance of animal and human health under a One Health framework is crucial for understanding and mitigating the impact of pathogenic viruses, it is important to consider that some viruses may have co-evolved with Antarctic fauna. These viruses may not be recent introductions or pathogens but part of the region’s complex microbial ecosystem (3, 8).

Seabirds are infected by a variety of microorganisms including protozoa, bacteria, and viruses (9–12). Although previous studies have demonstrated the presence of a variety of viruses affecting the Antarctic avifauna (13–17), our comprehension of the endemic viral diversity in this region, which includes potentially zoonotic pathogens, remains limited. Among viruses infecting seabirds, avian influenza viruses (AIVs) and coronaviruses (CoVs) warrant special attention due to their capacity to cause high mortality in both wild and domestic birds, resulting in significant population-level impacts (6, 18, 19).

Coronaviruses are classified into four genera based on their phylogenetic relationships: *Alphacoronavirus*, *Betacoronavirus, Gammacoronavirus*, and *Deltacoronavirus*. Alpha and Beta coronaviruses primarily infect mammals, while Gamma and Delta coronaviruses primarily infect birds, although mammal infection has also been reported (20). Despite coronaviruses being known to circulate in wildlife populations, there is limited investigation of coronaviruses in Antarctic fauna. In the absence of more extensive monitoring programs, it is not possible to determine which viral species circulate, which animals are potential reservoirs, the associated spillover risks, and what is the prevalence or burden of these viruses in animal hosts (7).

Wild birds (mainly Anseriformes and Charadriiformes) are recognized as the natural reservoir of AIVs (21, 22). It is estimated that 100 million seabirds live in the Antarctic Peninsula and adjacent islands, regularly encountering migratory birds that use the islands for nesting (1, 23). The ecology and migratory patterns of these birds probably have a direct effect on the global distribution and diversity of AIVs (13, 14). Especially concerning is the recent spread of highly pathogenic influenza A subtype of the H5N1 strain to the Antarctic continent (6, 24).

To fill the surveillance gaps about CoV and AIV viruses in Antarctic wildlife, we performed targeted viral surveillance for these viruses in fresh fecal samples from various Antarctic bird species sampled at the South Shetland Archipelago during the austral summer of 2023. We detected a single fecal sample positive for a *Deltacoronavirus* and recovered its full genome. This virus belongs to the *Buldecovirus* subgenus and is clustered with a full genome from a *Deltacoronavirus* circulating in the same region detected in the gentoo penguin (*Pygoscelis papua*) sampled in 2014, supporting long-term maintenance of the virus in the region and a potential cross-species transmission event or a broad host range of this virus.

## RESULTS

**Fig 1:**
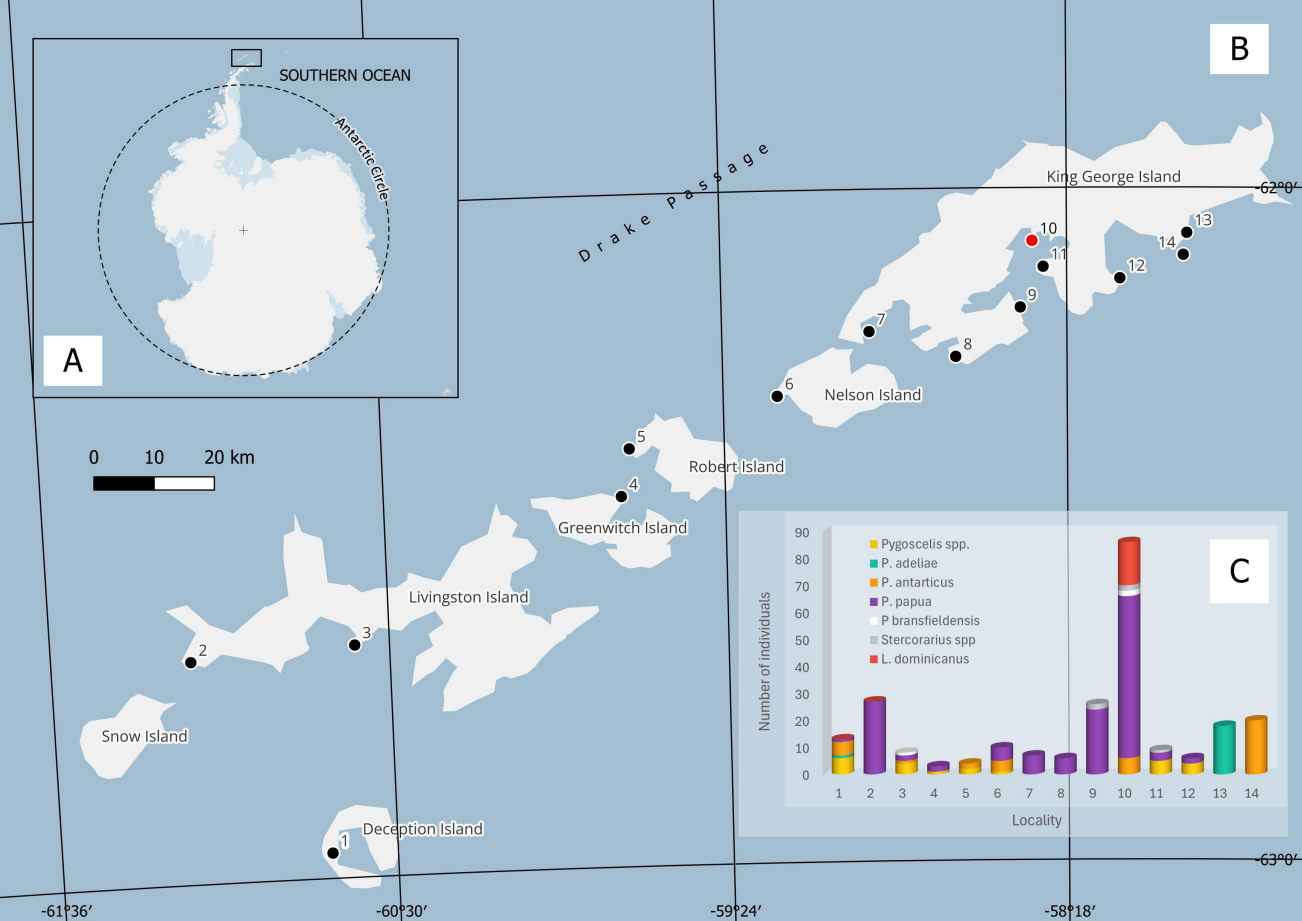
(**A**) Localization of the South Shetland Islands collection sites in the present study. (**B**) Localization of the sampling sites along the South Shetland Islands in January–February 2023. 1—Fumarole Bay, 2—Byers Peninsula, 3—Hannah Point, 4—Maldonado Base, 5—Coppermine Peninsula, 6—Harmony Point, 7—Ardley Island, 8—Potter Peninsula, 9—Copacabana, 10—Keller Peninsula, 11—Point Hennequin, 12—Lions Rump, 13—Three Sisters Point. In red, marked where the novel coronavirus was found. (**C**) Number of samples collected from each sampling area per species. More details in Table S1. Map made using a package from the platform Quantarctica 3.2 database in the software QGIS 3.32.2.

During the Brazilian Antarctica Expedition in January–February 2023, 243 fresh fecal avian samples were collected from skuas (*Stercorarius* spp.) (*n* = 5), kelp gulls (*Larus dominicanus*) (*n* = 16), Antarctic shags (*Phalacrocorax bransfieldensis*) (*n* = 3), Adélie penguin (*Pygoscelis adeliae*) (*n*= 19), chinstrap penguin (*Pygoscelis antarcticus*) (*n* = 38), gentoo penguin (*P. papua*) (*n* = 139), and not identified penguin species from the genus *Pygoscelis* spp. (*n* = 23) (Fig. 1C; Table S1). All tested samples were negative for avian influenza viruses (AIV), and one sample from the colony of *L. dominicanus* at Keller Peninsula, King George Island (Fig. 1B), tested positive for CoVs (sample F1596). The observed birds did not show any signs indicating disease.

**Fig 2:**
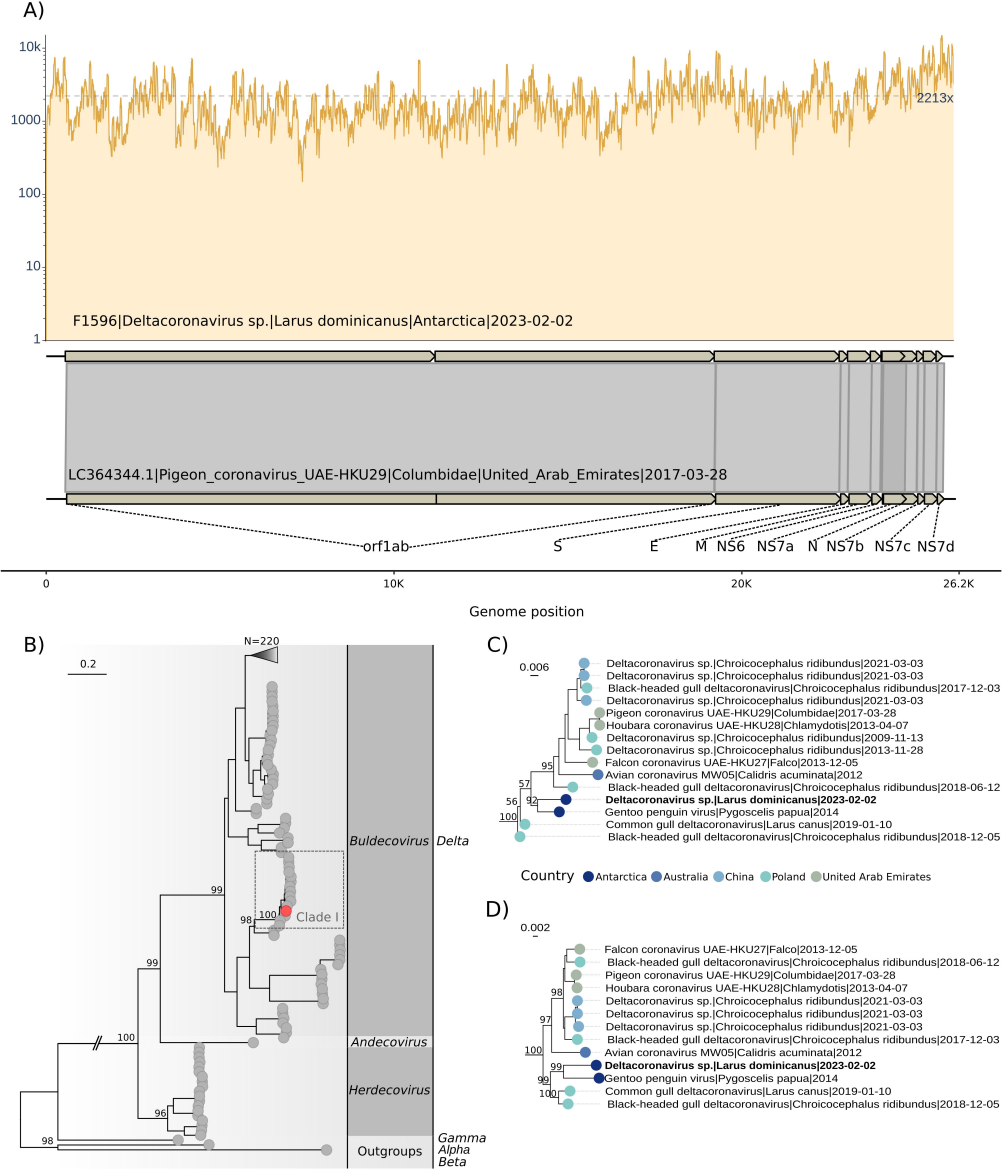
Genomic map and phylogenetic analysis of *Deltacoronavirus* identified in *Larus dominicanus*. (**A**) Genomic map showing the coverage of *Deltacoronavirus* F1596 and comparison with the best annotated reference (LC364344.1). (**B**) Phylogenetic analysis of nucleotide RdRp alignment of deltacoronaviruses using TIM2+F+I+G4 evolutionary model. The outgroups represent sequences from Alpha (OQ540913.1)*,* Beta (LC706864.1), and Gamma (MN509588.1) coronaviruses. Red tip point shows the positioning of the DeltaCoV F1596. (**C**) Zoom of clade I. (**D**) Clade I reconstructed based on phylogenetic analysis of amino acid ORF1ab-polyprotein alignment using Q.plant+F+I+G4 evolutionary model. The phylogenetic trees were reconstructed on IQ-TREE 2.3.6 (25) performing the ultrafast bootstrap with 1,000 replicates. Bold tip labels represent the sequence characterized in this study. Tip point colors represent the sampling location.

A total of 64.2 million (32.1 of paired-end) Illumina reads were generated for *L. dominicanus* sample, and 52.6 million quality-filtered reads were assembled into 7,292 contigs. One contig of 26,093 base pairs (bp) and mean depth coverage of 2240.26 x was assembled and identified as a complete genome of a *Deltacoronavirus*. This virus was designated DeltaCoV/AvCoV/Larus_dominicanus/Antarctica/Fiocruz-F1596/2023 (Fig. 2A). The assembled genome has been deposited in GenBank under PQ351810.1 accession number.

**Fig 3:**
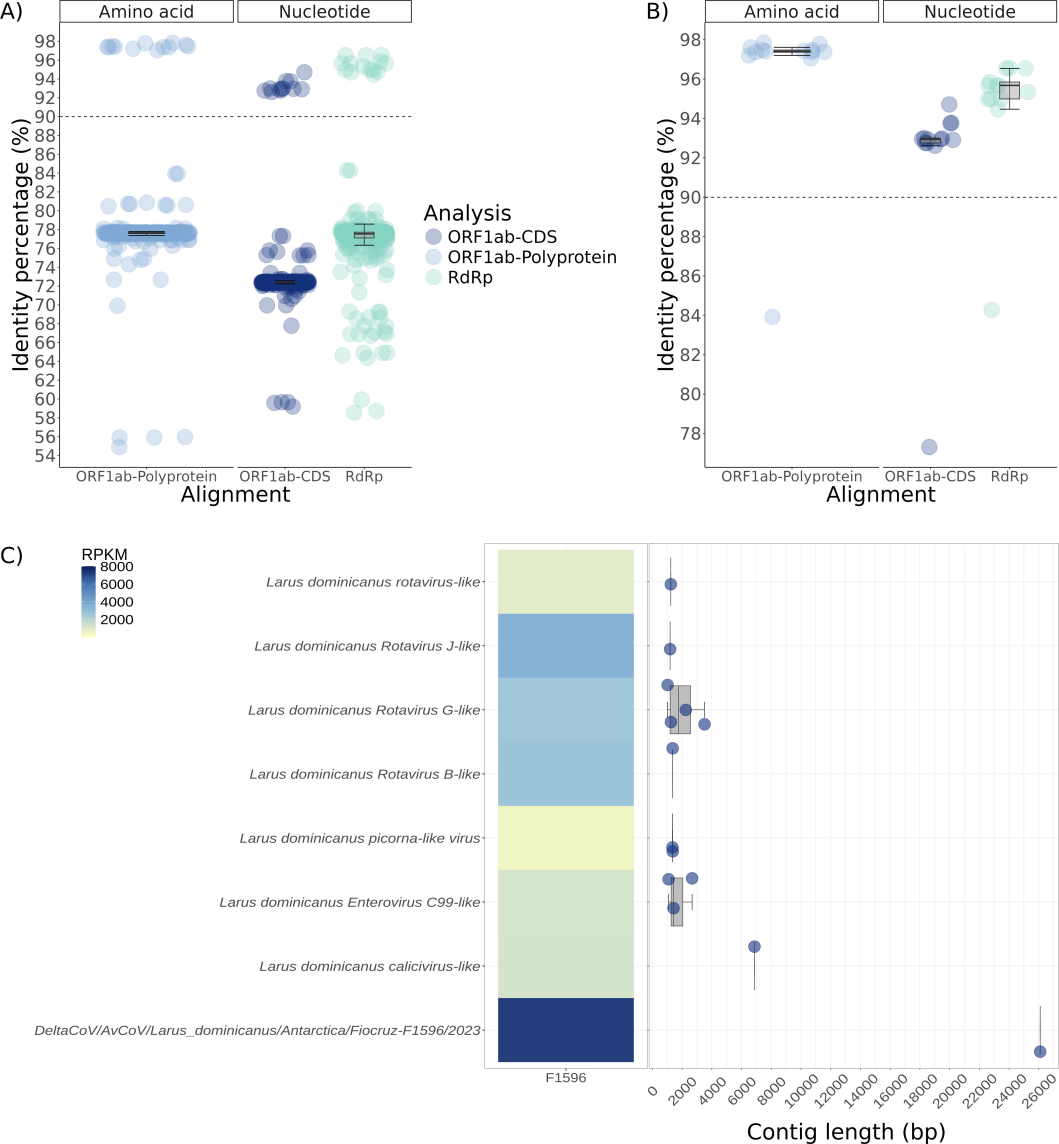
Genetic distances of *Deltacoronavirus* identified in *Larus dominicanus* and those from NCBI, reads per kilobase per million (RPKM) abundance and contig length of viruses identified. (**A**) Distances among deltacoronaviruses from the clade depicted in Fig. 2B. (**B**) Distances among deltacoronaviruses from the clade depicted in Fig. 2C. (**C**) Heatmap showing the RPKM abundance and contig length of viruses identified in this study.

The pairwise homoplasy index (Phi) test for recombination showed no significant values for recombination on nucleotide alignments. Therefore, three maximum likelihood phylogenetic analyses were performed based on alignments of RdRp nucleotide region, ORF1ab-CDS and polyprotein encoded by ORF1ab. The RdRp phylogenetic analysis, which included 316 coronaviruses (313 Delta, 1 Alpha, 1 Beta, and 1 Gamma coronaviruses), positioned the *Deltacoronavirus* from *L. dominicanus* within the *Buldecovirus* subgenus shown in clade I (Fig. 2B). Clade I comprises 15 *Buldecovirus* sequences identified in Antarctica, Australia, China, Poland, and the United Arab Emirates from other gulls (Fig. 2C). *Larus dominicanus Buldecovirus* sequence was closely related to gentoo penguin virus detected in *P. papua* identified in Antarctica, Isla Kopaitik, and Base O'Higgin in 2014 (MT025058.1) (26) with a high node support of 92 (ultrafast bootstrap) in the RdRp phylogeny. This finding was corroborated by the analysis of ORF1ab-CDS (Fig. S1) and polyprotein sequence encoded by ORF1ab, which showed high node supports of 99 and 100 (ultrafast bootstrap), respectively (Fig. 2D). The identity percentages between the Deltacoronavirus identified in L. dominicanus and other sequences from the NCBI database varied as follows: for nucleotide ORF1ab-CDS alignment, they ranged from 59% to 94% (median = 72.38, standard deviation = 4.64); for ORF1ab-polyprotein alignment, from 54% to 97% (median = 77.65, standard deviation = 5.03); and for nucleotide RdRp alignment, from 58% to 96% (median = 77.54, standard deviation = 5.05) (Fig. 3A and B; Tables S2 and S3). The *Deltacoronavirus* identified in *L. dominicanus* showed identities of 96.54, 94.71, and 97.61% (Table S2) with the sequences from gentoo penguin virus based on nucleotide RdRp, ORF1ab-CDS, and ORF1ab-polyprotein alignments. Both viruses are closely related to those found primarily in Charadriiformes birds from distant geographical locations (Fig. S2).

In addition to the *Deltacoronavirus* identified in this study, our analysis identified another 13 viral contigs showing best pairwise-alignment hits with four viral taxa (*Sedoreoviridae, Picornavirales, Picornaviridae*, and *Caliciviridae*) (Fig. 3C). We identified seven *Rotavirus* sequences, two picorna-like virus sequences, three *Enterovirus* C99-like virus sequences, and one calicivirus-like sequence. The coverM analysis showed the *Deltacoronavirus* as the most abundant virus, followed by the *Rotavirus* (Fig. 3C). The amino acid identities of these viruses in relation to their best hits ranged from 37% to 62% for *Rotavirus*, 42% to 62% for picorna-like viruses, 94% to 97% for *Enterovirus* C99-like viruses, and 40% for calicivirus-like sequence (Table S4).

## DISCUSSION

Coronaviruses have a global distribution and can be found in various species of wild and domestic animals, serving as natural reservoir hosts. Virus evolution in coronaviruses may happen through the acquisition of new genetic mutations by accruing single amino acid changes as well as through recombination events (20). Deltacoronaviruses are commonly found in wild birds and have been detected in all continents, including Antarctica (17, 18, 27). The deltacoronaviruses currently contain seven ratified species that belong to three subgenera, all of which have been detected in wild birds (28, 29). Deltacoronaviruses appear to be the most prevalent in Anseriformes and Charadriiformes; however, their diversity in wild birds is likely much broader than currently documented. According to a recent review, avian coronaviruses have been detected in 15 orders, comprising 30 families, and across 108 species of wild birds (27) across a wide range of host species, including gulls, shorebirds, penguins, passerines, and even bustards (17, 18, 27, 30).

The strain detected in our study was closely related to gentoo penguin virus identified in *P. papua* from Antarctica, Isla Kopaitik, and Base O'Higgin in 2014, but in locations 140 km apart (26), suggesting long-term maintenance of this *Deltacoronavirus* lineage in Antarctica and potential cross-species transmission and/or long-term infection of multiple avian species. According to the International Committee on Taxonomy of Viruses (ICTV; https://ictv.global/report_9th/RNApos/Nidovirales/Coronaviridae), viruses that share more than 90% aa sequence identity in the conserved replicase domains are considered to belong to the same species; thus the DeltaCoV identified belongs to a potential novel viral species that is still not ratified by the ICTV, but that was tentatively named novel wild birds DeltaCoV clade (27). The same authors suggested that this gentoo penguin virus could spill over from other birds such as skuas (*Stercorarius* spp.) or gulls inhabiting the same islands (26). Our results bring new evidence that supports cross-species transmission of this *Deltacoronavirus* between species that inhabit the Antarctic environment. Although the virus spread may be simultaneously taking place through interspecies spillover and penguin interpopulation migrations. *L. dominicanus* distribution overlaps with that of gentoo penguins, providing opportunities for interspecies viral transmission (Fig. S2). Moreover, this species has generalist and opportunistic feeding behaviors, frequently scavenging and preying within penguin colonies (31, 32). This carrion and eggs feeding behaviour may facilitate *L. dominicanus* infection from infected penguin juveniles (33). However, it remains to be evaluated whether the DeltaCoV detected in *L. dominicanus* feces can actively infect *L. dominicanus,* or if its presence is due to excreta from gentoo penguin-infected juveniles. Moreover, the alternative hypothesis of long-term maintenance of a multi-host *Deltacoronavirus* should also be further evaluated to fully understand the host range and the inter- and intra-species transmission dynamics of this virus in natural settings.

Approximately 40 species of sea birds breed on the Antarctic Peninsula, adjacent islands, and the Antarctic mainland (1). The kelp gull is widely distributed across the Southern Hemisphere, nests in South America, southern Africa, Australia, New Zealand, sub-Antarctic islands, and the Antarctic Peninsula, including Keller Peninsula on the southern side of King George Island (34, 35). During the austral winter, most Antarctic populations leave their breeding grounds and disperse across marine environments in the Southern Hemisphere, including Australia, New Zealand, South Africa, and South America (36). This migration provides an opportunity for the spread or emergence of new viruses, as the movement of *L. dominicanus* between regions could facilitate viral transmission across diverse ecosystems. In addition, all Laridae species live in colonies with high densities, where contact between infected individuals can easily occur, and these factors may favor the infection of gulls with various coronaviruses (37, 38).

We acknowledge the limitations of our study, as the fecal samples were collected from the environment rather than directly from the birds and could represent environmental contamination. However, most of the samples were collected shortly after the time of defecation. Additionally, the samples from the gull colony were taken in an area exclusively inhabited by gulls, with no penguin colonies present on Keller Peninsula. Penguins are only seen sporadically along the shoreline. Additionally, one would expect that environmental contamination with viral particles or viral genome fragments would result in viral genomes sharded on many contigs with low coverage depth appearing in multiple fecal samples from the same colony. However, we only detected a DeltaCoV in one single sample and recovered its full genome with high genome coverage depth, which is indicative of high viral load being excreted by feces likely from an active infection. Therefore, we can confidently attribute the fecal samples to gulls rather than other bird species and infer infection of this species by the *Deltacoronavirus* detected here.

The AIVs not only circulate in humans but also in domestic animals such as pigs, horses, and poultry, as well as in wild migratory birds, where more than 100 species of ducks, geese, swans, gulls, waders, and wild aquatic birds are considered natural reservoirs (22). Numerous studies have investigated the occurrence of AIVs in the avifauna of Antarctica, consistently showing that the local fauna is constantly exposed to AIV infections (13, 14, 39–43). However, all tested fecal samples were negative during monitoring carried out by our group in 2023, similarly to findings obtained in the same breeding season on the South Shetland Islands by Muñoz et al. (44) and in 2022 by Gomes et al. (45). Therefore, these aggregated results suggest an absence of AIV infection in Antarctic birds, corroborating the literature data that have consistently observed a low prevalence (rates typically below 5%) of infection caused by AIVs in Antarctic seabirds (13, 14, 26, 40, 43). Despite that, in October 2023 (next breeding season after our study), the first case of highly pathogenic avian influenza was detected among the birds of the Antarctic region for the first time, emphasizing that continuous monitoring and surveillance of influenza viruses among Antarctic fauna is warranted (6).

Lastly, as metatranscriptomic approaches uncover the full breadth of viruses of any given sample (mostly viruses with RNA genome but also viruses with DNA genome), we detected and assembled full and partial virus genomes from four families known to infect other bird species. These data further support that the Antarctica fauna harbors a diverse set of viruses that have been evolving with their host for a long period of time (15, 26). Moreover, such rich genomic data will be key to charactere the current circulating viruses in Antarctica, the introduction of new viruses and their threats to wildlife.

### Conclusions

We successfully detected a novel *Buldecovirus* in the feces of *L. dominicanus* and sequenced its complete genome. This virus is closely related to a DeltaCov previously identified in *P. papua* from the Antarctic Peninsula. Although no AIVs were found, our analysis revealed additional viral contigs, which showed the highest sequence similarity to four viral taxa: *Sedoreoviridae*, *Picornavirales*, *Picornaviridae*, and *Caliciviridae*. This underscores the vast, largely unexplored viral diversity in Antarctic ecosystems. Given the limited and fragmented understanding of the ecology of AIVs and CoVs in these remote environments, our findings emphasize the critical need for ongoing surveillance and research. Such efforts are vital to elucidate the dynamics of AIV circulation in Antarctic wildlife, ensuring the protection of both animal and human health.

## MATERIALS AND METHODS

### Sample collection

Fecal samples were collected during field expeditions conducted in the South Shetland Islands, near the Antarctic Peninsula, in January and February 2023. The sampling efforts covered 8 islands and 14 different localities (Fig. 1). During the sampling process, we collected individual fresh samples from monitored animals as well as fecal material from penguins’ nesting sites. Fecal samples were obtained using sterile Dacron swabs, which were immediately placed into tubes containing 1 mL of Viral Transport Medium composed of DMEM Cell Culture Medium, supplemented with fetal bovine serum (10%), and antibiotics and antifungals (penicillin 100 IU/ml, streptomycin 50 µg/mL, amphotericin B 0.1 µl/mL, gentamicin 1,000 µg/mL, and kanamycin sulfate 650 µg/mL) (41). Subsequently, the samples were refrigerated for a maximum of 4 hours before being frozen at −80°C for subsequent analysis.

### Viral RNA extraction

Clarified fecal suspensions (20% wt/vol) were prepared as previously described (41). Subsequently, 140 µL of the supernatant was utilized for viral RNA extraction. The extraction process was conducted using a QIAamp viral RNA mini kit (Qiagen, CA, USA) following the manufacturer’s guidelines. The isolated RNA was promptly stored at −80°C until further molecular analysis. Negative controls for each extraction procedure were carried out using RNase/DNase-free water.

### CoVs screening by conventional pancoronavirus RT-PCR

All the samples were also subjected to pancoronavirus PCR targeting the RNA-dependent RNA polymerase (*RdRp*) gene as described previously (18, 45, 46). In brief, RNA was first amplified using a first-round PCR (RdRp S1 5′-GGKTGGGAYTAYCCKAARTG-3′, RdRp R1 5′-TGYTGTSWRCARAAYTCRTG-3′) with the One-Step RT-PCR Enzyme MixKit (Qiagen), targeting a total expected size of 602 bp. Subsequently, a second PCR was performed using the Phusion RT-PCR Enzyme Mix kit (Sigma-Aldrich) and primers Bat1F 5′-GGTTGGGACTATCCTAAGTGTGA-3′ and Bat1R 5′-CCATCATCAGATAGAATCATCAT-3′, with 1 µL of the first-round amplified product as template. The resulting amplicons (~440 bp) were visualized on 1.5% agarose gels stained with SYBR Safe DNA Gel Stain (Thermo Fisher Scientific). For Sanger sequencing, DNA was purified using the QIAquick Gel Extraction Kit (Qiagen) following the manufacturer’s recommendations. The Sanger sequencing reaction was prepared utilizing the BigDye Terminator v3.1 Cycle Sequencing Kit (Life Technologies) with primers Bat1F and Bat1R at a concentration of 3.2 pmoles. Sequencing was executed on the ABI 3730 DNA Analyzer (Applied Biosystems) following the protocols established by (47). The final consensus sequences were derived using Sequencher 5.1.

### AIV screening by quantitative one-step real-time RT-PCR

The screening for AIVs was conducted utilizing the gene M qRT-PCR kit (Biomanguinhos, Fiocruz). Samples exhibiting a characteristic sigmoid curve and crossing the threshold line below a threshold cycle (CT) value of 38 were considered positive.

### Metatranscriptomic sequencing

After validation of *Deltacoronavirus* detection in the F1596 sample by Sanger sequencing, we processed the same sample by metatranscriptomics with the goal of recovering the full *Deltacoronavirus* genome. The positive sample was treated with the Ambion TURBO DNA-free Kit (Thermo Fisher Scientific, Waltham, MA, USA) to remove residual genomic DNA from the extracted RNA. Initially, 30 µL of RNA was combined with 3 µL of 10× TURBO DNase Buffer and 1 µL of TURBO DNase in a microcentrifuge tube. The mixture was incubated at 37°C for 30 minutes to allow the DNase to degrade any contaminating DNA. Following incubation, 3 µL of stop solution was added, and the sample was further incubated for 5 minutes at room temperature to ensure complete inactivation of the DNase. The sample was then centrifuged at 10,000 × g for 1.5 minutes, and the supernatant was transferred to a new tube. To deplete host rRNA and prepare RNA for sequencing, we used the Illumina Stranded Total RNA Prep, Ligation with Ribo-Zero Plus kit (Illumina, San Diego, CA, USA), which integrates rRNA depletion with library preparation. This kit includes enzymatic fragmentation, adapter ligation, and strand-specific cDNA synthesis, enabling efficient construction of sequencing libraries for Illumina platforms. Libraries were sequenced on the Illumina NextSeq platform (Illumina, San Diego, CA, USA) using a NextSeq 1000/2000 P2 cartridge.

### *Deltacoronavirus* genome characterization and phylogenetic analysis

Raw reads generated from NextSeq Illumina sequencing were first analyzed on fastp v0.23.2 to trim low-quality reads with a cutoff of 20 for Phred score and a minimum read length of 36 bp. Trimmed reads were used to perform a *de novo* assembly analysis on metaSPAdes v3.15.5 in default mode (48). All contigs were submitted to a Diamond blastx analysis (49) against a custom data set composed of all viral proteins assigned to taxonomy tag (txid10239) on NCBI plus RdRp sequence databases such as NeoRdRp (50), PalmDB (51), and RdRp-Scan (52) as performed previously by da Silva et al. (53). The sequences showing the best hits on viral sequences were submitted to a second Diamond blastx analysis against the non-redundant database from National Center for Biotechnology Information (NCBI) to remove false negative hits. Additional BLASTn (54) analyses were conducted against the NT database from NCBI. Only contigs showing hits against viral proteins in these two analyses were kept and further analyzed. Contigs annotated as viral were analyzed on the ViralComplete (55) tool to classify them into full-length or partial according to their completeness in relation to NCBI RefSeq genomes. The trimmed reads were then mapped against the contigs using Bowtie2 in default mode. The BAM file was sorted and indexed by Samtools 1.20 (56), and genome metrics were calculated using the CoverM tool (https://github.com/wwood/CoverM). Genomic maps were constructed using the BAMdash tool (https://github.com/jonas-fuchs/BAMdash) and *gggenomes* R package (https://github.com/thackl/gggenomes).

For the phylogenetic analysis, we downloaded all closely related *Deltacoronavirus* sequences from NCBI virus that matched the F1596 *Deltacoronavirus* in a blastn analysis implemented within the NCBI database (https://blast.ncbi.nlm.nih.gov/Blast.cgi). All *Deltacoronavirus* RdRp sequences from NCBI virus were also included in our analysis. Additionally, we included reference sequences from Alpha (OQ540913.1)*,* Beta (LC706864.1), and Gamma (MN509588.1) coronaviruses to root our trees. All retrieved sequences were aligned together with the sequenced genome using MAFFT v7.511. Nucleotide alignments were inspected for recombination using SplitsTree App v.6.3.35 running the Phi test (57). For the phylogenetic analysis, we performed three approaches: (i) nucleotide alignment of partial RdRp sequences, (ii) nucleotide CDS alignment of ORF1ab, and (iii) translated CDS amino acid sequences of ORF1ab. The maximum likelihood phylogenetic analysis was performed on IQ-TREE 2.3.6 with the SH-aLRT test and ultrafast bootstrap with 1000 replicates. The best evolutionary models were selected by ModelFinder (58). The trees were visualized and annotated using the *ggtree* R package (59). Distance matrices were calculated using the alignments in MEGA 11.0.13, performing a p-distance method and rates among sites parameter set as G4+I and gaps/missing data treated with pairwise deletion.

To better understand and visualize potential virus transmission between species, we mapped the distributions of bird species in which closely related coronaviruses were detected (belonging to the new *Deltacoronavirus* clade), using data from the BirdLife database (60).

## Data Availability

The raw reads were submitted to NCBI Sequence Read Archive (SRA) database and are available under project number: PRJNA1160912 and Run Accessions: SRR30664529 and SRR30664530.

## SUPPLEMENTAL MATERIAL

Fig. S1 spectrum.02688-24-s0001.jpg ORF1ab-CDS phylogeny of *Deltacoronavirus* identified in *Larus dominicanus*.

Fig. S2 spectrum.02688-24-s0002.jpg Distributions of *Larus dominicanus*, *Pygoscelis papua*, and bird species in which coronaviruses closely related to ours were detected, using data from the BirdLife database (BirdLife, 2023).

Supplemental material spectrum.02688-24-s0003.docx Legends.

Table S1 spectrum.02688-24-s0004.xlsx Localization of the collection sites in the present study and the number of bird fecal samples collected, South Shetland Islands, January-February 2023.

Table S2 spectrum.02688-24-s0005.xlsx Distance analysis of deltacoronavirus from *L. dominicanus* and deltacoronaviruses from GenBank.

Table S3 spectrum.02688-24-s0006.xlsx Statistics from distance analysis.

Table S4 spectrum.02688-24-s0007.xlsx The amino acid identities of detected viruses in relation to their best hits.

ASM does not own the copyrights to Supplemental Material that may be linked to, or accessed through, an article. The authors have granted ASM a non-exclusive, world-wide license to publish the Supplemental Material files. Please contact the corresponding author directly for reuse.
