# Supplementary figures and images for: Long-term maintenance of a Deltacoronavirus infecting multiple bird species in Antarctica
Source: Microbiol Spectr. 2025 Jun 16;13(8):e02688-24. doi: 10.1128/spectrum.02688-24 (PMC12323614; doi:10.1128/spectrum.02688-24)

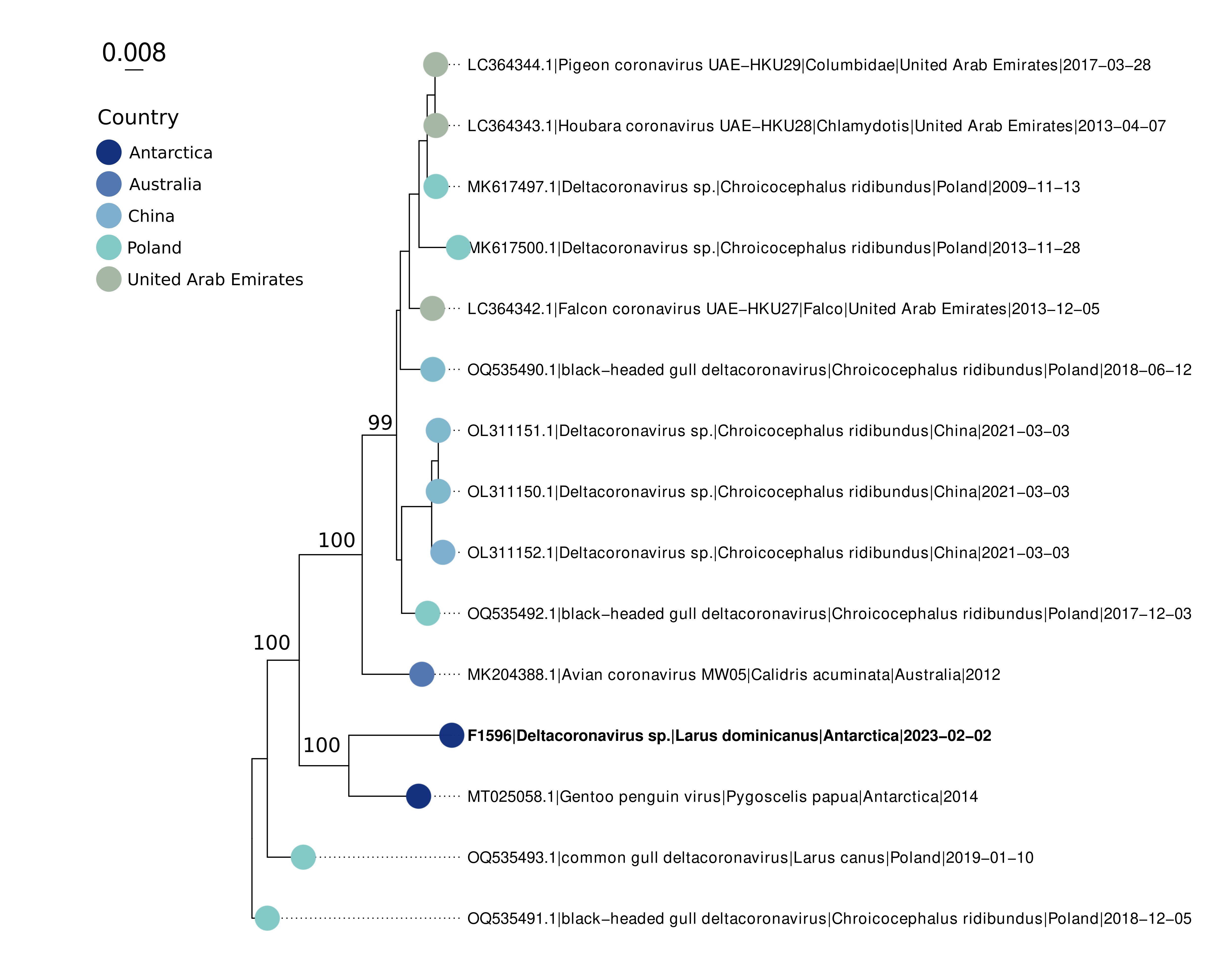

Supplement: Fig. S1 — ORF1ab-CDS phylogeny of Deltacoronavirus identified in Larus dominicanus. [file spectrum.02688-24-s0001.jpg]

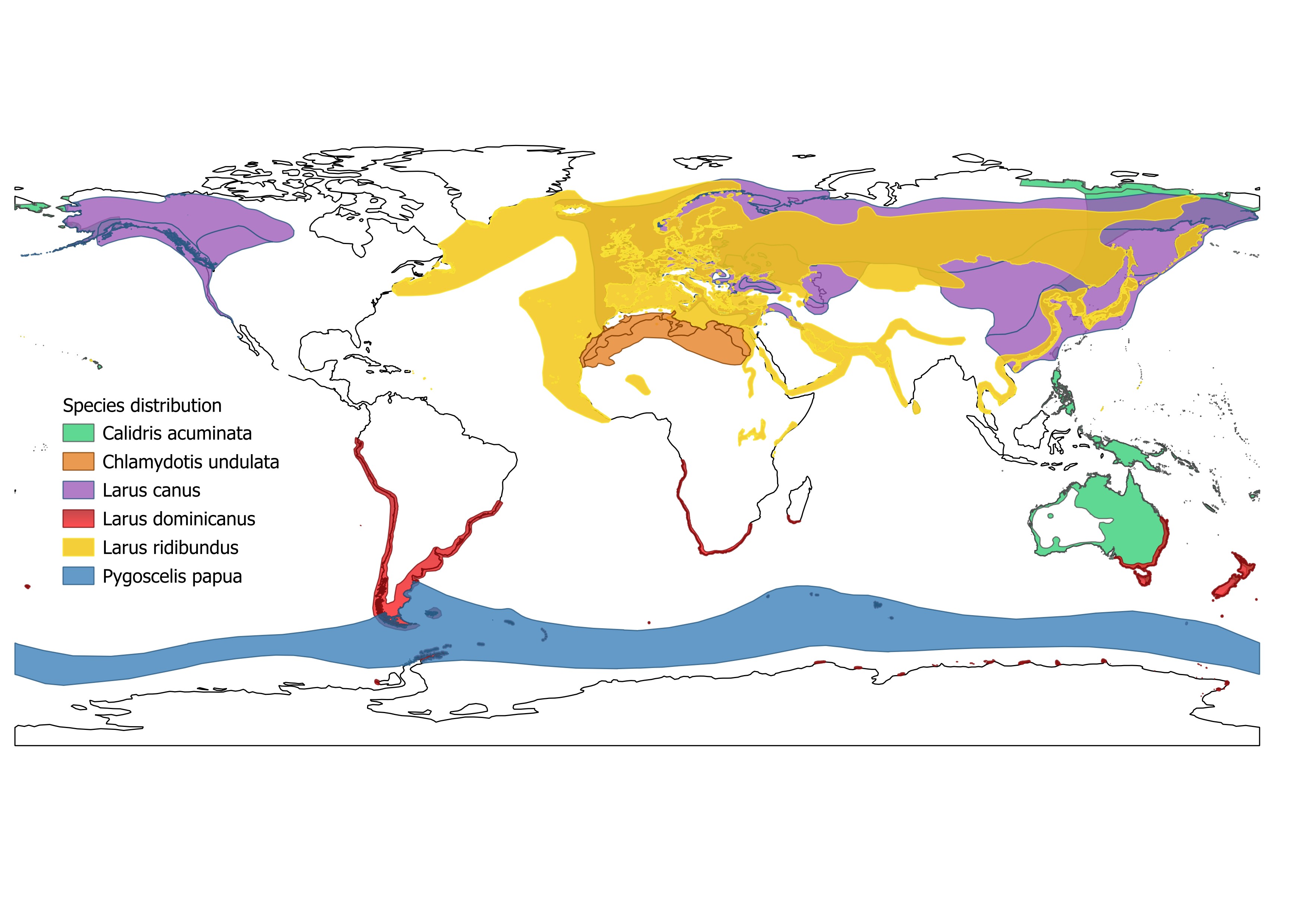

Supplement: Fig. S2 — Distributions of Larus dominicanus, Pygoscelis papua, and bird species in which coronaviruses closely related to ours were detected, using data from the BirdLife database (BirdLife, 2023). [file spectrum.02688-24-s0002.jpg]
