# Supplementary material for: Long-term maintenance of a Deltacoronavirus infecting multiple bird species in Antarctica
Source: Microbiol Spectr. 2025 Jun 16;13(8):e02688-24. doi: 10.1128/spectrum.02688-24 (PMC12323614; doi:10.1128/spectrum.02688-24)
Supplement: Supplemental material — Legends. [file spectrum.02688-24-s0003.docx]

**Supplementary material legends**

**Supplementary figure 1.** ORF1ab-CDS phylogeny of *Deltacoronavirus* identified in *Larus dominicanus*. Phylogenetic tree showing the clade I based. The tree was reconstructed on IQ-TREE2.3.6 using the GTR+F+I+G4 evolutionary model.

**Supplementary figure 2 –** Distributions of *Larus dominicanus*, *Pygoscelis papua* and bird species in which coronaviruses closely related to ours were detected, using data from the BirdLife database (BirdLife, 2023). The map made in QGIS 3.32.2 (QGIS, 2024).

**Supplementary table 1.** Localization of the collection sites in the present study and the number of bird fecal samples collected, South Shetland Islands, January-February 2023. ASPA - Antarctica Specially Protected Area.

**Supplementary table 2 -** Distance analysis of *Deltacoronavirus* from *Larus. dominicanus* and deltacoronaviruses from GenBank.

**Supplementary table 3.** Statistics from distance analysis.

**Supplementary table 4 -** The amino acid identities of detected viruses in relation to their best hits**.**
